# The intensity of a resistance exercise session can be quantified by the work rate of exercise

**DOI:** 10.1371/journal.pone.0291857

**Published:** 2023-10-05

**Authors:** Brendan R. Scott, Kieran J. Marston, Shaun Y. M. Teo, Mitchell R. L. Forrest, Andrew Jonson, Thomas P. Walden, Brook Galna, Jeremiah J. Peiffer

**Affiliations:** 1 Centre for Healthy Ageing, Murdoch University, Murdoch, Australia; 2 Murdoch Applied Sports Science Laboratory, Discipline of Exercise Science, Murdoch University, Murdoch, Australia; 3 Telethon Kids Institute, Perth Children’s Hospital, Nedlands, Australia; 4 Allied Health Department, Fiona Stanley Hospital, Murdoch, Australia; 5 Discipline of Exercise Science, College of Science, Health, Engineering and Education, Murdoch University, Murdoch, Australia; Universidade Estadual Paulista Julio de Mesquita Filho - Campus de Bauru, BRAZIL

## Abstract

**Purpose:**

Athletes regularly perform resistance training, yet it is unknown how best to monitor its intensity. This study compared different resistance exercise intensity metrics to determine their sensitivity to manipulating work rate (via altering inter-set rest and load).

**Methods:**

Following baseline testing for 10- and 3-repetition maximum (RM; squat and bench press), fourteen trained participants completed four volume-matched protocols in a randomised order: 3x10 with 85% 10RM, 60 s rest (3x10_60s_); 3x10 with 85% 10RM, 180 s (3x10_180s_); 8x3 with 85% 3RM, 120 s (8x3_120s_); 8x3 with 85% 3RM, 300 s (8x3_300s_). Internal intensity was quantified via rate of oxygen consumption ($\dot{V}O_{2}$), heart rate, blood lactate concentration, and rating of perceived exertion (RPE). External intensity was assessed via previously developed “Training-Intensity” (TI) and “Intensity-Index” (II) metrics, and from exercise work rate (expressed as kg∙min^-1^ and joules∙min^-1^).

**Results:**

Internal intensity and work-rate metrics were highest for 3x10_60s_, followed by 3x10_180s_, 8x3_120s_ and 8x3_300s_ (p≤0.027). TI and II were higher for 8x3 than 3x10 protocols (p<0.001), but not different within these configurations. Internal intensity measures were more strongly correlated with work rate (*r* = 0.37–0.96) than TI and II (*r* = -0.42–0.33) metrics.

**Conclusions:**

Work rate corroborated objective internal intensity metrics during resistance exercise, with the highest work rate session (3x10_60s_) also eliciting greater RPE scores than other protocols. In contrast, the TI and II did not agree with other intensity measures, likely because they do not consider rest periods. Practitioners can plan for the physiological and perceptual demands of resistance training by estimating work rate.

## Introduction

Resistance training is commonly prescribed to improve physical performance and/or attenuate injury risk [1]. The calculation of training loads, simply defined as the product of exercise volume and intensity [2], can enhance the understanding of how an individual responds to resistance exercise. The volume of resistance training is measured as the amount of work accomplished [3, 4], or estimated as the volume load (sets × repetitions × load lifted) [3]. Quantifying resistance exercise intensity is more complex as it represents the mechanical, physiological, and psychological demands of exercise [5, 6], and can be broken down further into internal and external components. Internal intensity refers to physiological or psychological stress during exercise [7], and can be determined via metabolic and cardiovascular responses [8] or rating of perceived exertion (RPE) [9]. External intensity refers to the rate at which work is completed [10]; however, this is difficult to assess in real-world training environments where direct measurement of work may not be feasible.

The previously developed “Training-Intensity” (TI) metric estimates resistance exercise intensity as the average load lifted across a particular exercise or training session (i.e., volume load / repetitions) [3, 10]. Further to this, the “Intensity Index” (II) can be calculated as the relative volume load (volume load / body mass) divided by the number of repetitions [3]. However, TI and II do not account for the inter-set rest periods during resistance exercise, which affects the internal intensity of a session [11]. The “work rate” of resistance exercise has been proposed to measure external intensity while accounting for variations in rest periods and exercise duration [6, 12–14]. This measure has been defined as the total work per unit time [14], and is calculated by dividing the total work or volume load (as a surrogate of work) by the session duration [13] or summed duration of inter-set rest periods [15]. When calculated directly from measured work, this external intensity metric is synonymous with mean power output across an entire exercise session [16]. Resistance exercise at a higher work rate elevates the acute physiological and psychosocial response (e.g., catecholamine levels, blood lactate, growth hormone, session RPE [sRPE]) and stimulates positive longer term muscular adaptations (e.g., muscle cross-sectional area, strength, and endurance) [13–15, 17]. However, if not monitored appropriately, extended periods of exercise at greater intensity, and the associated metabolic demand as seen in resistance exercise at an increased work rate, may increase an individual’s risk of staleness, burnout or overtraining syndrome [18]. Unfortunately, few practical methods of measuring work rate are currently available for resistance training; instead, alternative metrics are preferred to quantify training loads (i.e., volume load, TI, II). Moreover, how well the current methods of measuring work rate relate to a robust array of physiological (e.g., change in blood lactate, heart rate and rate of oxygen consumption) and psychosocial (e.g., sRPE) outcomes also remains unknown.

Considering the extensive implementation of resistance training for clinical, healthy, and athletic populations, it is important to understand the influence of manipulating work rate (via changes to acute exercise variables) on indices of internal and external exercise intensity. Therefore, the aim of this study was to investigate metrics of resistance exercise intensity, by assessing whether they can discriminate between exercise sessions purposely designed to have different work rates. We hypothesised that greater work rate during resistance exercise would translate to higher internal intensity, whereas TI and II metrics would not reflect other intensity measures.

## Materials and methods

### Participants

Seven males (23.7 ± 3.3 yr; 176.9 ± 7.7 cm; 83.8 ± 10.1 kg) and seven females (23.2 ± 2.7 yr; 165.7 ± 6.9 cm; 62.9 ± 6.4 kg) participated in this study. Participants were recruited via advertisements posted at the university and snowball sampling. All participants had >2 years resistance training experience, reported no use of substances that could affect the study’s results, and were instructed to avoid strenuous exercise for 24 hours prior to each testing session. Participants were provided with information detailing the purpose and requirements of the research, gave signed informed consent, and were screened for medical contraindications. The study and its methods were approved by the Institutional Human Research Ethics Committee (2017/204).

### Design

Participants visited the laboratory twice for baseline testing of their 3RM and 10RM in the free-weight back squat and bench press, using established methods [19]. Attempts were made at increasingly heavy loads until participants could not successfully complete a set (e.g., inability to complete a repetition, failure to achieve full range of motion or if technique deteriorated and was deemed unsafe). The 3RM and 10RM was defined as the heaviest completed set, which was determined within 3–6 sets separated by three minutes rest.

**Fig 1:**
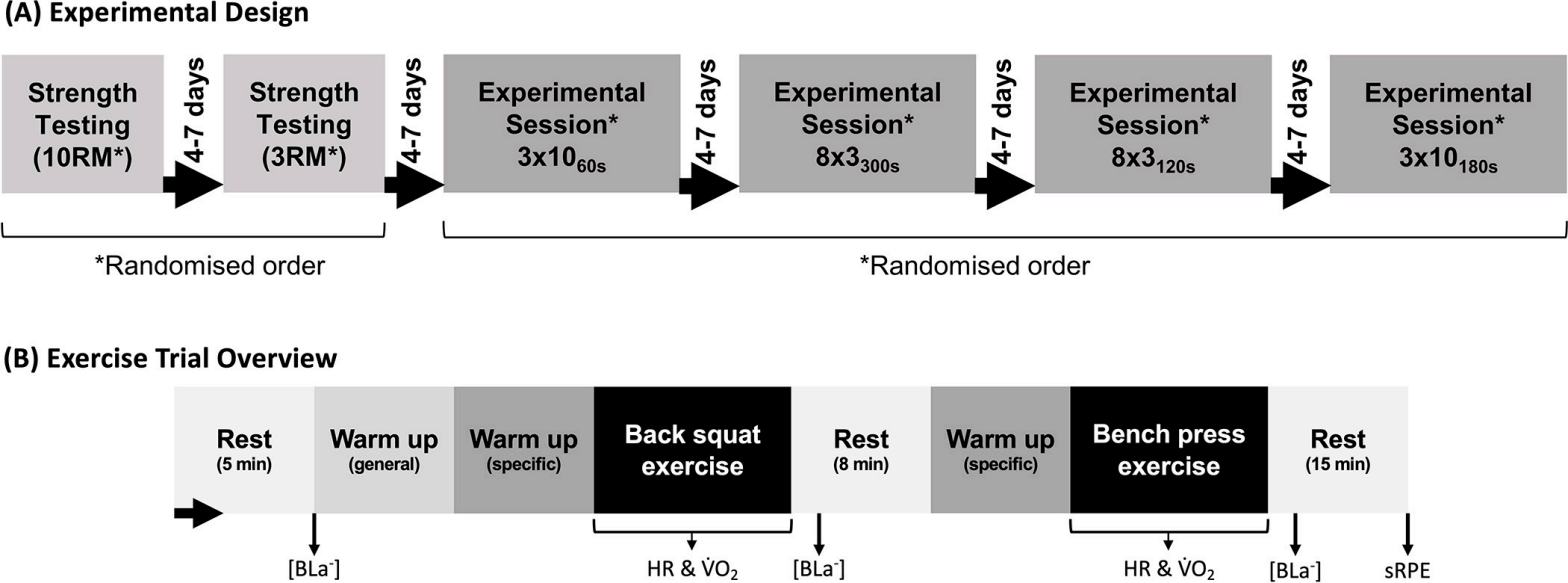
Summary of the (A) study structure from repetition maximum (RM) assessment to experimental sessions, and (B) structure of individual experimental sessions, including the timing of internal intensity measures. RM = repetition maximum, [BLa^-^] = blood lactate concentration, HR = heart rate, $\dot{V}O_{2}$, = rate of oxygen consumption, sRPE = session rating of perceived exertion.

Following a within-subject design, participants then completed four different exercise protocols in a randomised order, separated by 4–7 days (Fig 1). Randomisation was completed via a random number generator by a researcher blinded to the participants’ identity. Trials were matched for the relative effort necessary to lift different loads (i.e., sets of 10 performed with 85% 10RM, sets of 3 with 85% 3RM), but varied in the absolute load being lifted and/or the inter-set recovery time: A) 3x10 with 85% 10RM and 60 s inter-set rest (3x10_60s_), B) 3x10 with 85% 10RM and 180 s rest (3x10_180s_), C) 8x3 with 85% 3RM and 120 s rest (8x3_120s_), and D) 8x3 with 85% 3RM and 300 s rest (8x3_300s_). These protocols represent ecologically valid examples of exercise prescription with different loads [19], yet were estimated *a priori* to require different work rates. Sessions were also matched as closely as possible for volume load. Internal exercise intensity was quantified via objective (rate of oxygen consumption [$\dot{V}O_{2}$], heart rate and blood lactate concentration) and subjective (set and session RPE) measures. External intensity was quantified via the TI and II metrics [10], and from the session work rate. The work rate was estimated from volume load (represented as kg∙min^-1^) and calculated from work (represented as joules∙min^-1^ to provide continuity with volume load-based work rate).

### Methodology

#### Experimental testing sessions

For experimental trials, participants were fitted with a face mask connected to a metabolic cart (TrueOne 2400, ParvoMedics, Sandy, USA) and a heart rate strap (Edge 500, Garmin, USA), before resting passively for 5 minutes. They then commenced 5 minutes self-paced cycling and specific warm-up sets for the squat (unloaded barbell [10 repetitions], 50% [7 repetitions for 3RM-based sessions or 10 repetitions for 10RM-based sessions], 70% [5 or 7 repetitions] and 90% [3 or 5 repetitions]) of RM load), before beginning the assigned exercise protocol. To encourage consistent effort between trials, participants were instructed to perform the eccentric phase of each repetition “under control” and the concentric phase “as fast as possible” [20]. After the final set of squats, participants rested for 8 minutes before performing the specific warm-up and working sets for the bench press. During these experimental sessions, internal and external measures of exercise intensity were recorded.

#### Measures of internal intensity

Objective internal exercise intensity was determined via the mean $\dot{V}O_{2}$ (ml∙kg^-1^∙min^-1^) and heart rate (beats∙min^-1^) values from the start of the first to the end of the last working set for each exercise, including inter-set rest. Additionally, blood lactate concentration was measured in duplicate from a fingertip at baseline (after the 5-minute rest), and at 2 minutes following the final set of each exercise. Capillary blood samples (0.7 μL) were analysed for lactate concentration using a hand-held analyzer (Lactate Plus, Nova Biomedical^®^, USA), with the mean of duplicate measures calculated for analysis. Subjective ratings of intensity were obtained via RPE scores, collected immediately following each set (reported as mean set RPE) and at 20 minutes following the conclusion of each trial (i.e., sRPE), using the Category Ratio-10 RPE scale [21].

#### Measures of external intensity

The external intensity of each exercise protocol was calculated using several methods. The TI metric was determined as the average load lifted for the squat and the bench press separately [10]. The II metric was calculated as volume index (i.e., volume load / body mass) divided by the number of repetitions [3]. Work-rate metrics were calculated by dividing the accumulated volume load (kg∙min^-1^) and work (joules∙min^-1^) for each exercise by the time taken from the start of the first set to the conclusion of the last set. This technique provided a practical approach to estimating work rate from volume load, as well as by assessing work during exercise. Work was assessed via a linear position transducer (GymAware, Kinetic Performance Technology, Canberra, Australia), which was attached to the barbell.

### Statistical analyses

Data were assessed for normality via the Shapiro-Wilk test and were normally distributed except for RPE data. Due to equipment malfunction, heart rate data were unavailable for six participants, and these data were analysed with the remaining subset of eight. Dependent variables (volume load, total work, TI, II, work rate, $\dot{V}O_{2}$, heart rate) were compared between experimental sessions separately for each exercise using linear mixed models, where session was included as a fixed effect and participants were modelled using random intercepts. Blood lactate concentration was also assessed via linear mixed models, using a 4 x 3 design with session, time (i.e., baseline, post-squat and post-bench press time points) and session x time included as fixed effects and participants were modelled using random intercepts. Pseudo marginal and conditional R^2^ values were calculated for each linear mixed model to indicate the proportion of total variance (variance due to fixed, random and error) explained by the fixed effects alone (marginal R^2^) and combination of fixed and random effects (conditional R^2^). Estimated marginal means were also calculated for each model, along with 95% confidence intervals about the mean. Where a significant main effect or interaction was observed, Fisher’s LSD *post hoc* assessment was used to identify where differences occurred. Friedman’s ANOVA was used to compare RPE scores separately for each exercise between trials, with Wilcoxon *post hoc*. Effect sizes were calculated for comparisons as Cohen’s *d*_*z*_ (difference in the mean divided by SD of the difference) [22].

Pearson’s product-moment correlations were calculated to assess the relationships between objective internal intensity and the external intensity metrics for each participant, while relationships between subjective internal intensity and external intensity were assessed via Spearman’s rho. The mean correlation coefficient and 95% confidence intervals were calculated after individual coefficients were Fisher Z transformed. Correlation coefficients were interpreted as trivial (0.00–0.09), small (0.10–0.29), moderate (0.30–0.49), large (0.50–0.69), very large (0.70–0.89), and near perfect (0.90–1.00) [23]. To avoid violating assumptions of independence, correlations were calculated between intensity measures for each participant across the four sessions and two exercises, with the mean ± SD and range for these group data determined. Data were analysed using SPSS (v27, Chicago, IL, United States), with statistical significance set at *p* ≤ 0.05, and data represented as mean ± SD unless stated otherwise.

## Results

The 3RM for the squat and bench press were 127.0 ± 16.6 kg and 95.6 ± 16.8 kg for males, and 72.4 ± 21.7 kg and 37.3 ± 7.4 kg for females. The 10RM for the squat and bench press were 103.1 ± 14.2 kg and 80.1 ± 14.8 kg for males, and 61.6 ± 18.3 kg and 31.9 ± 6.3 kg for females. Volume load did not differ between the 3x10 (squat: 2100.0 ± 679.3 kg, bench press: 1435.7 ± 696.9 kg) and 8x3 (squat: 2036.6 ± 689.9 kg, bench press: 1378.3 ± 653.7 kg) protocols for either exercise (p = 0.970). Work also was not different between all four protocols (squat: 21.0 ± 6.4 to 23.9 ± 6.7 kJ, bench press: 5.2 ± 2.4 to 5.5 ± 2.5 kJ; p ≥ 0.542).

**Fig 2:**
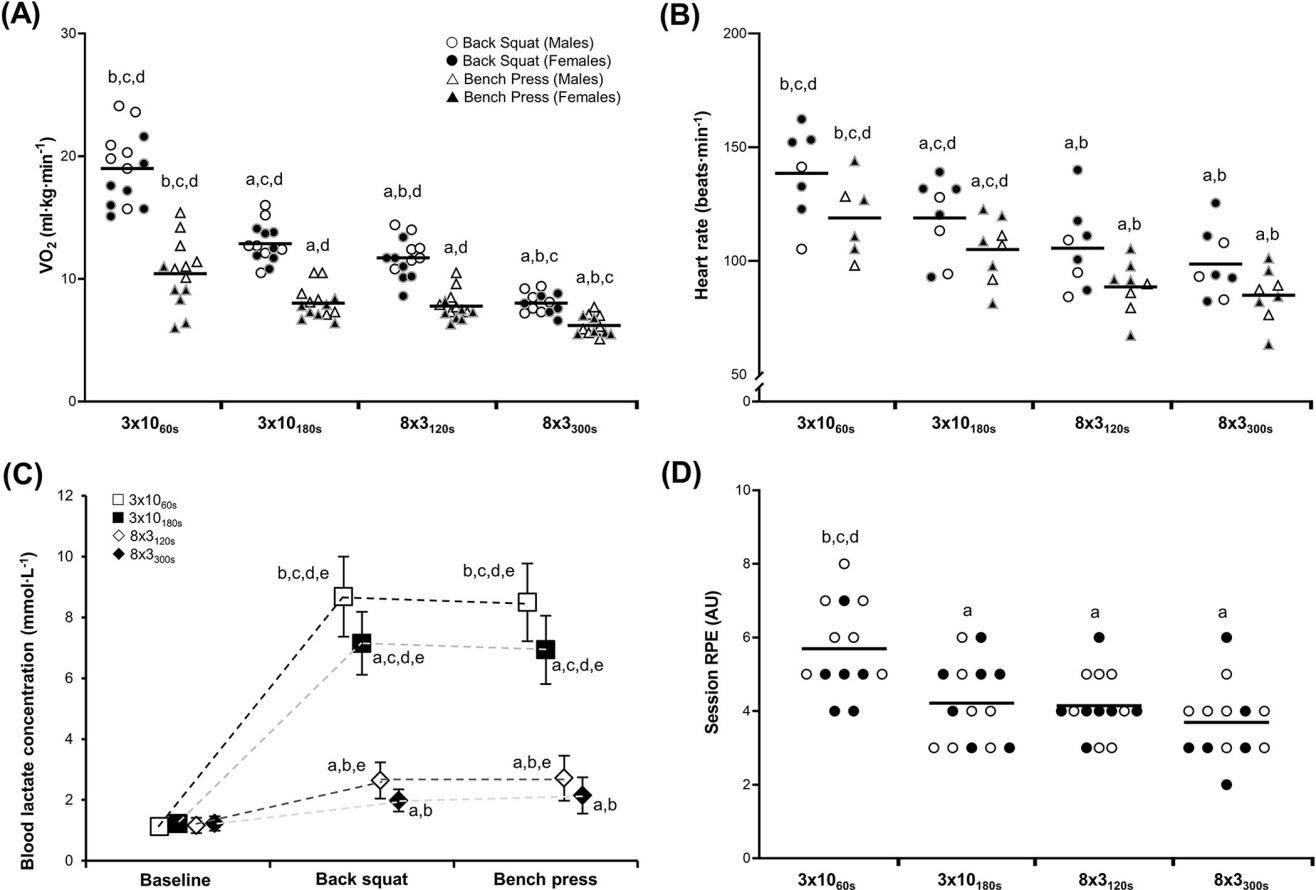
Internal intensity during resistance exercise protocols, represented as (A) mean rate of oxygen consumption ($\dot{V}O_{2}$), (B) mean heart rate, (C) blood lactate concentration, and (D) session rating of perceived exertion (RPE). Mean and 95% confidence intervals shown. a = different to 3x10_60s_, b = different to 3x10_180s_, c = different to 8x3_120s_, d = different to 8x3_300s_, e = increased from baseline. **Note:** Some data points are missing at random due to equipment or collection errors; individual data reported for transparency.

Internal measures of exercise intensity are shown in Fig 2 and Tables 1–3. Significant main effects of session were observed for $\dot{V}O_{2}$ in both exercises (p < 0.001). $\dot{V}O_{2}$ was highest for 3x10_60s_ sessions, followed by the 3x10_180s_, 8x3_120s_ and 8x3_300s_ sessions (p ≤ 0.015, *d*_*z*_: 1.2–5.0), except for between the 3x10_180s_ and 8x3_120s_ in the bench press (p = 0.561, *d*_*z*_: 0.4). Significant main effects for session were also observed for heart rate in both exercises (p < 0.001), with highest values observed for the 3x10_60s_ sessions, followed by the 3x10_180s_, 8x3_120s_ and 8x3_300s_ (p ≤ 0.006, *d*_*z*_: 1.3–5.0). However, the 8x3_120s_ and 8x3_300s_ were not different for either exercise (p ≥ 0.068, *d*_*z*_: 0.6–0.8).

**Table 1:** Mean (95% confidence intervals) for oxygen consumption ($\dot{V}O_{2}$) and heart rate during sessions and exercise type.

	Session	Back squat	Bench press
$\dot{V}O_{2}$	3x10_60s_	19.0 (18.0, 20.0) ^b^^,^^c^^,^^d^	10.4 (9.5, 11.3) ^b^^,^^c^^,^
(ml∙kg^-1^∙min^-1^)	3x10_180s_	12.9 (11.8, 13.9) ^a^^,^^c^^,^^d^	8.0 (7.1, 8.9)^a^^,^^d^
	8x3_120s_	11.7 (10.7, 12.8) ^a^^,^ ^b^^,d^	7.8 (6.9, 8.7) ^a^^,d^
	8x3_300s_	7.9 (6.8, 9) ^a^^,^^b^^,c^	6.1 (5.2, 7.0) ^a^^,^^b^^,c^
	R^2^	Marginal: 0.82, Conditional: 0.93	Marginal: 0.46, Conditional: 0.76
**Heart rate**	3x10_60s_	140 (126, 154)^b^^,^^c^^,^^d^	116 (104, 127) ^b^^,^^c^^,^
(beats·min^-1^)	3x10_180s_	119 (105, 133) ^a^^,^^c^^,^^d^	105 (94, 116) ^a^^,^^c^^,^^d^
	8x3_120s_	106 (92, 120) ^a^^,^^b^	89 (78, 100) ^a^^,^^b^
	8x3_300s_	99 (85, 113) ^a^^,^^b^	85 (74, 96) ^a^^,^^b^
** **	R^2^	Marginal: 0.44, Conditional: 0.90	Marginal: 0.44, Conditional: 0.89

a = different to 3x10_60s_

b = different to 3x10_180s_

c = different to 8x3_120s_

d = different to 8x3_300s_.

**Table 2:** Mean (95% confidence intervals) blood lactate concentration (mmol·L^-1^) for each session and exercise.

Session	Baseline	Back squat	Bench press
3x10_60s_	1.1 (0.3, 1.9)	8.7 (7.9, 9.5) ^b^^,^^c^^,^^d^^,^^e^	8.5 (7.7, 9.3) ^b^^,^^c^^,^^d^^,^^e^
3x10_180s_	1.2 (0.4, 2.0)	7.1 (6.4, 7.9) ^a^^,^^c^^,^^d^^,^^e^	6.9 (6.2, 7.7) ^a^^,^^c^^,^^d^^,^^e^
8x3_120s_	1.2 (0.4, 2.0)	2.6 (1.9, 3.4) ^a^^,^^b^^,^^e^	2.7 (1.9, 3.5) ^a^^,^^b^^,^^e^
8x3_300s_	1.2 (0.4, 2.0)	1.9 (1.1, 2.7) ^a^^,^^b^	2.1 (1.3, 2.9) ^a^^,^^b^
	Marginal R^2^: 0.80, Conditional R^2^: 0.86		

a = different to 3x10_60s_

b = different to 3x10_180s_

c = different to 8x3_120s_

d = different to 8x3_300s_

e = increased from baseline.

**Table 3:** Median (25^th^ percentile, 75^th^ percentile) session rating of perceived exertion (RPE) during each experimental trial.

Session	Session RPE (AU)
3x10_60s_	5.00 (5.00, 7.00)^b^^,^^c^^,^^d^
3x10_180s_	4.00 (3.00, 5.00)^a^
8x3_120s_	4.00 (3.75, 5.00)^a^
8x3_300s_	4.00 (3.00, 4.00)^a^

a = different to 3x10_60s_

b = different to 3x10_180s_

c = different to 8x3_120s_

d = different to 8x3_300s_.

A significant session x time interaction was observed for blood lactate concentration (p < 0.001), with values increasing from baseline in all sessions (p ≤ 0.002, *d*_*z*_: 1.1–2.9) except for the 8x3_300s_ (p ≥ 0.061, *d*_*z*_: 0.9–1.3). The highest post-exercise blood lactate values were observed in the 3x10_60s_ sessions, followed by the 3x10_180s_, 8x3_120s_ and finally the 8x3_300s_ session, with significant differences between each trial (p ≤ 0.002, *d*_*z*_: 0.9–3.9), except for between the 8x3_120s_ and 8x3_300s_ sessions (p ≥ 0.140, *d*_*z*_: 0.4–0.7).

A significant main effect was observed for session in sRPE (p = 0.003), with post hoc analyses confirming higher scores for the 3x10_60s_ session compared with other trials (all p ≤ 0.010, *d*_*z*_: 1.1–1.3). For mean set RPE scores (not shown in figure), a significant effect of session was observed for the squat (p = 0.020) and bench press (p = 0.009). In the squat, mean set RPE was higher in the 3x10_60s_ session (RPE = 5.0 ± 1.1) than the 8x3_300s_ (RPE = 4.0 ± 1.1; p ≤ 0.027, *d*_*z*_: 0.8). For the bench press, higher values were recorded in the 3x10_60s_ session (RPE = 4.8 ± 0.8) than the 3x10_180s_ and 8x3_300s_ sessions (RPE = 3.9 ± 0.8 and 3.7 ± 0.8, respectively; p ≤ 0.008, *d*_*z*_: 1.1–1.3).

**Fig 3:**
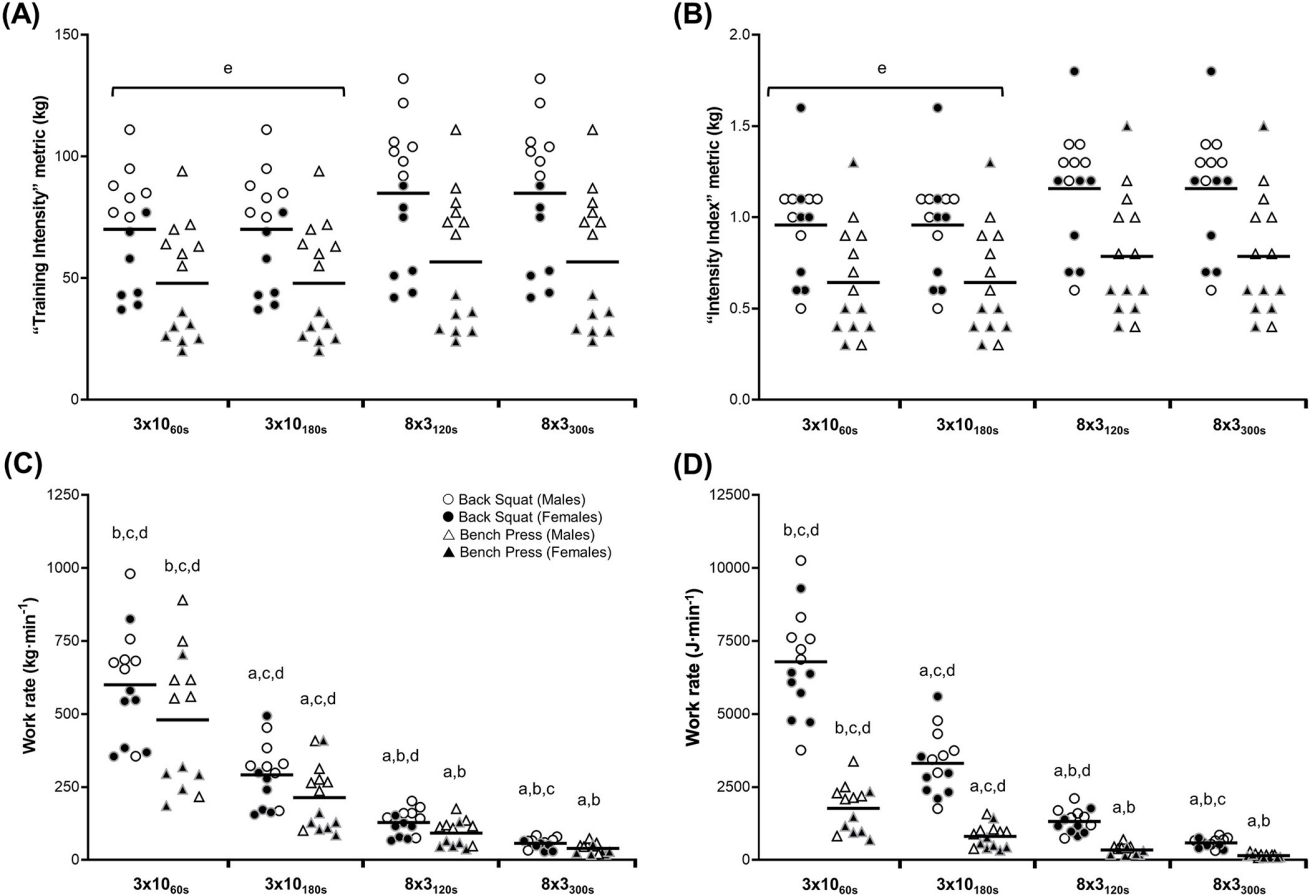
External intensity during resistance exercise protocols, represented as (A) “Training Intensity” metric, (B) “Intensity Index metric”, (C) work rate (kg∙min^-1^), and (D) work rate (J∙min^-1^). Mean and 95% confidence intervals shown. a = different to 3x10_60s_, b = different to 3x10_180s_, c = different to 8x3_120s_, d = different to 8x3_300s_, e = different to 8x3 protocols. **Note:** Some data points are missing at random due to equipment or collection errors; individual data reported for transparency.

External measures of exercise intensity are shown in Fig 3 and Table 4. For both the TI and II metrics, there were significant main effects for session (all p < 0.001), with lower values in the 3x10 compared with 8x3 sessions (p < 0.001, *d*_*z*_: 1.9–2.4). For work rate (kg∙min^-1^), significant main effects of session were observed for both exercises (p < 0.001). Higher values were observed in the 3x10_60s_ session, followed by the 3x10_180s_, 8x3_120s_ and finally the 8x3_300s_ session (all p ≤ 0.013, *d*_*z*_: 1.8–3.3), although the 8x3_120s_ and 8x3_300s_ sessions did not differ for the bench press (p = 0.145, *d*_*z*_: 2.2). Significant main effects of session were also observed for work rate (J∙min^-1^) in both exercises (p < 0.001). Higher values were observed in the 3x10_60s_ session, followed by the 3x10_180s_, 8x3_120s_ and finally the 8x3_300s_ session (p ≤ 0.009, *d*_*z*_: 1.9–4.1), although 8x3_120s_ and 8x3_300s_ in the bench press were not different (p = 0.113, *d*_*z*_:2.3).

**Table 4:** Mean (95% confidence intervals) external intensity for each session and exercise.

	Session	Back squat	Bench press
**Work rate**	3x10_60s_	600 (538, 661)	477 (404, 550)
(kg·min^-1^)	3x10_180s_	291 (229, 353)	213 (142, 285)
	8x3_120s_	128 (66, 190)	87 (14, 160)
	8x3_300s_	49 (0, 112)	31 (0, 104)
	R^2^	Marginal: 0.78, Conditional: 0.90	Marginal: 0.63, Conditional: 0.81
**Work rate**	3x10_60s_	6786 (6196, 7375)	1755 (1498, 2013)
(J·min^-1^)	3x10_180s_	3310 (2721, 3899)	803 (551, 1055)
	8x3_120s_	1318 (728, 1907)	328 (71, 585)
	8x3_300s_	518 (0, 1120)	114 (0, 371)
	R^2^	Marginal: 0.84, Conditional: 0.92	Marginal: 0.65, Conditional: 0.82
**“Training Intensity”**	3x10_60s_	70 (55, 85)	48 (33, 62)
(kg)	3x10_180s_	70 (55, 85)	48 (33, 62)
	8x3_120s_	85 (70, 100)	57 (43, 72)
	8x3_300s_	85 (70, 100)	57 (43, 72)
	R^2^	Marginal: 0.08, Conditional: 0.97	Marginal: 0.04, Conditional: 0.99
**“Intensity Index”**	3x10_60s_	0.96 (0.78, 1.13)	0.64 (0.46, 0.83)
(kg)	3x10_180s_	0.96 (0.78, 1.13)	0.64 (0.46, 0.83)
	8x3_120s_	1.16 (0.98, 1.33)	0.79 (0.6, 0.97)
	8x3_300s_	1.16 (0.98, 1.33)	0.79 (0.6, 0.97)
** **	R^2^	Marginal: 0.10, Conditional: 0.98	Marginal: 0.05, Conditional: 0.99

Correlations between internal and external intensity measures are displayed in Table 5. Internal intensity metrics were more strongly associated measures of work rate, than with TI and II.

**Table 5:** Mean (95% confidence intervals) and range of correlation coefficients across participants between internal and external training intensity measures. Scatterplots of the raw data for each participant is presented in S1 Fig.

	“Training Intensity” (kg)	“Intensity Index” (kg)	Work rate (kg·min^-1^)	Work rate (J·min^-1^)
$\dot{V}O_{2}$ (ml·kg·min^-1^)	0.33 (0.17, 0.46) range: -0.11, 0.71	0.33 (0.17, 0.46) range: -0.11, 0.71	0.84 (0.79, 0.88) range: 0.65, 0.95	0.96 (0.95, 0.97) range: 0.84, 0.98
**Heart rate** (beats·min^-1^)	0.20 (-0.04, 0.42) range: -0.26, 0.64	0.2 (-0.04, 0.42) range: -0.26, 0.64	0.91 (0.83, 0.96) range: 0.72, 0.99	0.89 (0.84, 0.93) range: 0.77, 0.95
**Blood lactate concentration** (mmol·L^-1^)	-0.42 (-0.55, -0.28) range: -0.75, -0.09	-0.42 (-0.55, -0.28) range: -0.75, -0.09	0.87 (0.82, 0.90) range: 0.64, 0.94	0.67 (0.62, 0.72) range: 0.53, 0.81
**Set RPE** (AU)	-0.03 (-0.20, 0.15) range: -0.70, 0.43	-0.03 (-0.20, 0.15) range: -0.70, 0.43	0.37 (0.13, 0.56) range: -0.53, 0.89	0.57 (0.20, 0.79) range: -0.49, 0.98

TI metric = training-intensity metric, II metric = intensity-index metric, $\dot{V}O_{2}$ = rate of oxygen consumption, RPE = rating of perceived exertion. Correlation coefficients calculated as Pearson’s except for those involving set RPE scores which are Spearman’s rho. Individual correlation coefficients were transformed prior to calculating means and confidence intervals using Fischer Z transformations and then back transformed for summary statistics presented in this table.

**NOTE:** Intensity Index is calculated as Training Index relative to body mass and so both exhibit identical correlations with internal intensity metrics. Correlations were calculated individually for each participant to avoid violating assumptions of independence.

## Discussion

The aim of this study was to investigate metrics of resistance exercise intensity and determine whether they can discriminate between exercise sessions designed to have different work rates (i.e., different prescription of the load lifted and/or inter-set rest periods). The main findings are: 1) objective measures of internal intensity ($\dot{V}O_{2}$, heart rate and blood lactate concentration) were highest for the protocols with the greatest work rate and lowest in those with lesser work rates, 2) subjective internal intensity was highest in the session with the greatest work rate, and 3) moderate to near perfect correlations were observed between work rate and internal exercise intensity, while the TI and II metrics only exhibited trivial to moderate relationships with internal intensity. These findings suggest that the external intensity of resistance exercise can be effectively calculated as work rate.

Internal intensity reflects how an individual responds to the stress of a training session in the context of their current state of preparation [24]. In this study, these individual responses were assessed via several objective ($\dot{V}O_{2}$, heart rate and blood lactate concentration) and subjective (sRPE) metrics. The highest objective internal intensity was recorded for the 3x10_60s_ session, followed by the 3x10_180s_, 8x3_120s_ and 8x3_300s_ protocols. In agreement with our hypothesis, these observations match the differences in work rate between protocols, which was highest in the 3x10_60s_ session and lowest for the 8x3_300s_. Our findings confer with previous research [15], which revealed that greater work rate increases metabolic responses [17]. Goto and colleagues [17] observed significant elevations in blood lactate concentration and growth hormone when a resistance exercise session is performed at a higher work rate, even when total volume is matched. In contrast, Hiscock et al. [25] did not observe higher blood lactate levels when work rate was increased. This may be explained by the low post-exercise blood lactate levels (1.6–2.0 mmol∙L^-1^) [25], perhaps due to the small muscle mass used during exercise (unilateral bicep curl) not eliciting large enough metabolic responses to differentiate between work rates. We observed large to near perfect (*r* = 0.66–0.95) correlations between work rate and objective internal intensity metrics, meaning that ~44–90% of the variance in objective internal intensity is explained by work rate. Our findings suggest that increasing the work rate of resistance exercise elicits greater acute physiological demands. Whilst there is only a small body of literature to examine the impact of exercise work rate on longer-term training adaptations, it is likely that work rate is an important variable for resistance training adaptations. In their study, Goto et al. had participants perform 12 weeks of resistance training matched for total volume, yet differing in work rate (i.e., without intra-set recovery vs. with intra-set recovery). Interestingly, the authors reported significantly greater maximal strength, quadriceps femoris cross-sectional area and muscular work capacity in participants who performed resistance training at a greater work rate than a lower work rate [17]. Although these findings are promising and highlight that a minimum required work rate is necessary for effective training adaptations, more research is needed to confirm the degree of influence that work rate has on longer term training outcomes.

Subjective internal intensity was also highest in the 3x10_60s_ session, though there were limited differences between the remaining protocols. Moderate relationships were observed between mean set RPE responses and work rate (*r*_*s*_ = 0.37–0.57; ~14–32% of variance explained). This is similar, albeit slightly weaker, to Hiscock et al. [13], who reported sRPE scores were moderately correlated (r = 0.45) with work rate across different resistance exercise structures. This discord between objective and subjective measures of intensity could suggests that set and sessional RPE scores are not sensitive enough to delineate between training configurations. However, an alternative explanation may be the deliberate matching of effort in each protocol caused similar perceptual responses; participants exercised at 85% of their RM range for all sessions. To illustrate, Kraft et al. [14] compared resistance exercise protocols matched for work rate but not relative effort (3x8 with 60% 1RM and 90 s, compared with 2x12 with 60% 1RM and 180 s rest), and suggested higher RPE in the 2x12 protocol was explained by greater work performed per set. However, one can extend on this to estimate via the Brzycki equation [26] that 60% of 1RM is ~86% of 12RM and ~74% of 8RM. It is therefore not surprising that the 2x12 protocol was rated more difficult by participants. While perceptual responses are simple to collect, future research is needed to elucidate how RPE is impacted by manipulations of resistance exercise.

The work rate calculations performed in this study provided a better reflection of internal intensity than the TI and II metrics. This is likely because the TI and II metrics do not incorporate inter-set rest periods [3], which is a primary determinant of intensity across an exercise session [11]. Indeed, when considering the 3x10 and 8x3 protocols separately, the TI and II are identical, whereas the internal intensity measures indicate higher demands with reduced rest durations. Further to this, comparisons of TI and II between the 3x10 and 8x3 protocols suggest that lifting heavier loads is more demanding, even when protocols are matched for volume load. The TI and II metrics seem to emphasise the heaviness of the load as the main driver of intensity, which is likely too simplistic [27] and not supported by other measures of external or internal intensity in our study. The TI and II metrics were also not strongly related to any internal intensity measures assessed in this study. Considering these findings, measures of external intensity which do not incorporate the duration of exercise and/or are focused primarily on the load lifted (e.g., TI and II) do not provide adequate assessment of the demands associated with resistance training.

While this study provides insights regarding the use of simple work-rate metrics to quantify resistance exercise intensity in young males and females, some limitations should be acknowledged. Due to equipment malfunction, small amounts of data were not collected; for example, we have presented heart rate data from a subset of our sample (n = 8). These heart rate responses provided similar trends to those from other internal intensity metrics, and we are therefore confident that this subset of participants is representative of the sample recruited. In addition, our findings may be specific to the exercises assessed; caution should be applied when comparing the intensity of single-joint with multi-joint exercises. It should also be acknowledged that alternative strategies to control the relative load lifted via measurement of concentric velocity [28] were not assessed in this research. We also selected not to control the cadence of exercise, but instructed participants to perform the eccentric phase in a controlled manner and the concentric phase as fast as possible. While this approach is representative of most real-world training contexts, altering the cadence of each repetition to manipulate the time under tension could also impact the measurements of intensity we examined, and this deserves future research. Finally, we did not assess each participant’s cardiovascular fitness at baseline, meaning it was not possible to report $\dot{V}O_{2}$ or heart rate data relative to individual maximums or thresholds. Inter-individual discrepancies in absolute cardiovascular fitness were accounted for by using a within-participant cross-over design, enabling us to compare these indices between exercise sessions.

### Practical applications

The simple estimation of work rate (volume load [kg] / exercise duration [minutes]) can accurately represent the external intensity of resistance exercise, which provides practitioners the ability to track individual responses to a given resistance training stimulus (e.g., via external:internal intensity ratios). This is important to measure, given that a higher work rate during resistance training may enhance longer-term muscular adaptations, but could also increase the risk of staleness, burnout or overtraining syndrome if programmed inadequately. External intensity metrics which do not consider the resistance exercise duration or rest periods, such as the “Training-Intensity” and “Intensity-Index” metrics, should not be used to reflect overall intensity. Subjective assessments of resistance exercise intensity may not be sensitive enough to delineate between different training structures if the variation is subtle.

### Conclusions

Calculating work rate from resistance exercise by measuring the work done or simply based on volume load, corroborated objective internal intensity metrics. The highest work rate session (3x10_60s_) was also rated perceptually as more intense that other protocols. In contrast to work rate, the TI and II external intensity metrics did not match internal intensity data, likely because they do not consider the inter-set rest periods.

## Supporting information

S1 Fig Scatterplots between measures of external and internal training intensity. Different colours represent different participants.
(JPG)
