# Supplementary figures and images for: The intensity of a resistance exercise session can be quantified by the work rate of exercise
Source: PLoS One. 2023 Oct 5;18(10):e0291857. doi: 10.1371/journal.pone.0291857 (PMC10553797; doi:10.1371/journal.pone.0291857)

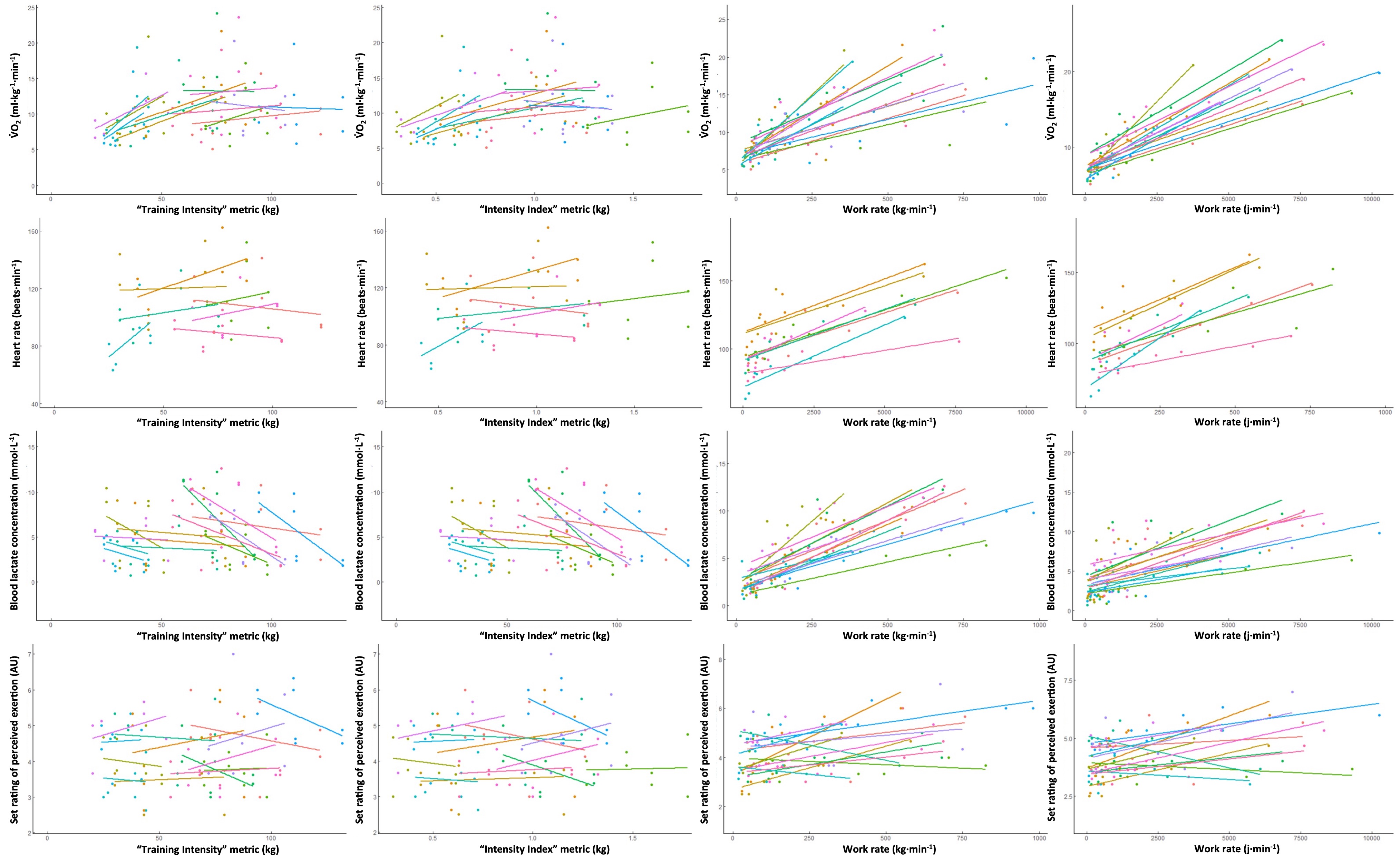

Supplement: S1 Fig — Different colours represent different participants. (JPG) [file pone.0291857.s001.jpg]
